# Association between Dietary Anthocyanidins and Risk of Lung Cancer

**DOI:** 10.3390/nu14132643

**Published:** 2022-06-26

**Authors:** Yin Zhang, Min Zhu, Huajing Wan, Ling Chen, Fengming Luo

**Affiliations:** 1 Department of Respiratory and Critical Care Medicine, West China Hospital, Sichuan University, Chengdu 610041, China; yinzhang9511@163.com (Y.Z.); huaxizhumin@wchscu.cn (M.Z.); wanhuajing1974@wchscu.cn (H.W.); cl2006190230@126.com (L.C.); 2 Laboratory of Pulmonary Immunology and Inflammation, Frontiers Science Center for Disease-Related Molecular Network, West China Hospital, Sichuan University, Chengdu 610041, China; 3 Clinical Research Center for Respiratory Disease, West China Hospital, Sichuan University, Chengdu 610041, China

**Keywords:** anthocyanidins, dose-response analysis, lung cancer

## Abstract

Background: Anthocyanidins are a kind of water-soluble flavonoids widely found in flowers and fruits of many plants. Although the beneficial effect of anthocyanidins in cancer prevention has been discussed, the value of anthocyanidins in lung cancer prevention requires further investigation. In this study, we aimed to explore the role of dietary anthocyanidins in the prevention of lung cancer in population-based prospective studies. Methods: Data of participants in this study were collected from the Prostate, Lung, Colorectal, and Ovarian Cancer Screening Trial. Hazard ratios (HRs) and 95% confidence intervals (CIs) were calculated in Cox proportional hazards regression for the association of dietary anthocyanidins and lung cancer risk. The dose-response relationship was explored between total anthocyanidins and the incidence of lung cancer. Results: A total of 97,993 participants were included in this study. The calculated HRs showed a trend that a higher quartile of total anthocyanidins indicated lower risk of lung cancer after adjusting for covariates (HR_Q4vsQ1_: 0.63; 95% CI: 0.55,0.73; *p* for trend < 0.001). A non-linear association between total anthocyanidins and lung cancer risk was found in the restricted cubic spline model. Conclusion: A protective association between dietary anthocyanidins and risk of lung cancer in Americans was investigated.

## 1. Introduction

The number of newly diagnosed lung cancer cases has risen for years. Causing an estimated 1.8 million deaths each year, lung cancer has been regarded as the leading cause of cancer deaths worldwide; thus, the prevention of lung cancer is an important research area [1,2]. In recent decades, the association between diet and the prevention of lung cancer has been discussed. Some healthy eating habits have been proven to be closely related to reduced lung cancer risk [3,4,5]. Anthocyanins are a kind of water-soluble flavonoids [6], the basic structures of which are anthocyanidins. In other words, anthocyanins are in the form of glycoside while anthocyanidins are in the form of aglycone [7]. The six main members of anthocyanidins are cyanidin, delphinidin, malvidin, peonidin, petunidin, and pelargonidin [7,8]. Commonly, the dietary sources of anthocyanidins include plants, especially flowers, fruits, and tubers containing a large amount of natural pigments [7]. Research shows that anthocyanidins participate in many health-promoting activities and have anti-oxidant, anti-inflammatory, anti-diabetic, anti-adipogenesis, and anti-cancer effects [9,10,11]. In particular, the beneficial effects of anthocyanidins in specific cancer prevention have been extensively discussed. Reviews have clearly demonstrated remarkable anti-cancer activity of anthocyanidins [12,13,14,15]. Anthocyanidins reduced the cell proliferation of tumors by blocking activation of the mitogen-activated protein kinase (MAPK) pathway [13]. In addition, they have exhibited anti-inflammatory effects in multiple cell types in vitro through inhibiting the expression of cyclooxygenase-2 (COX-2), NF-κB, and interleukins [13]. Other potential cancer chemopreventive activities of anthocyanidins include radical scavenging activity, stimulation of phase II detoxifying enzymes, angiogenesis and invasiveness, and induction of apoptosis and differentiation [13]. In animal models, anthocyanidins were proven to inhibit the development of tumors induced by subcutaneous injection of lung tumor cells in mice [16,17]. Disappointingly, these encouraging experimental findings in vitro and in animal models have not been extrapolated to existing human research to the same extent. Epidemiological studies in humans have not provided such convincing evidence of the anti-cancer effects of anthocyanidins [12,13]. A prospective study with 7534 postmenopausal women in the USA and another study with 2590 middle-aged eastern Finnish men showed that anthocyanidins were not significantly associated with lung cancer risk in women and men, respectively [18,19]. Therefore, the value of anthocyanidins in lung cancer prevention requires further investigation. In this study, we aimed to explore the role of dietary total anthocyanidins and the subclasses in the prevention of lung cancer in population-based prospective studies to add more evidence in this field.

## 2. Materials and Methods

### 2.1. Study Population

This study was approved by the United States National Cancer Institute (NCI) (CDAS project “PLCO-800”). Written informed consent to participate in the study was provided by each participant, and the study protocol was approved by the Institutional Review Board of the NCI (https://biometry.nci.nih.gov/cdas/plco/ accessed on 2 March 2022). Data in this study were collected from the Prostate, Lung, Colorectal, and Ovarian (PLCO) Cancer Screening Trial, a large-scale randomized clinical trial (RCT) designed and sponsored by the NCI to evaluate whether screening methods can reduce mortality from certain cancers in men and women aged 55 to 74. The PLCO trial was carried out at 10 centers in the United States from 1993 to 2001, enrolling over 154,000 healthy subjects. The participants who met the eligibility criteria were randomized to either the intervention group (received certain screening tests) or the control group (received usual care). All participants were asked to complete self-reported questionnaires about their lifestyle and were followed up with until 2009 for cancer incidence. The questionnaires included the baseline questionnaire (BQ), diet history questionnaire (DHQ), etc. [20]. The BQ was given to participants to collect baseline information at enrollment. The DHQ is a food frequency questionnaire (FFQ) containing dietary information, which evaluates the food or nutrient intake of each individual over the past year. Several researchers have assessed its validity, suggesting that DHQ is a good instrument for nutrient evaluation [21,22]. According to the objective of this study, we excluded subjects if they (1) failed to provide complete baseline information (including data needed to be extracted for this study, see Section 2.2); (2) failed to complete a valid DHQ (i.e., a date of completion was not available, the date of completion was not prior to the date of death, there were at least 8 missing frequency responses, or calorie intake was extreme (top 1% and bottom 1%) for each gender); or (3) had history of any cancer before DHQ entry.

### 2.2. Data Collection

Participants were arranged to complete a self-administered BQ containing personal baseline information. In this study, we collected trial arm (intervention or control), age, gender (male or female), baseline body mass index (BMI), race/ethnicity (white or non-white), marital status (married or not married), cigarette pack-years, family history of lung cancer (yes, no, or possible), and family history of any cancer (yes or no). The DHQ was used to collect the dietary information, including total daily energy intake, daily intake of anthocyanidins (including cyanidin, delphinidin, malvidin, pelargonidin, peonidin, and petunidin), and alcohol intake (never, former, current, or unknown).

### 2.3. Dietary Intake of Anthocyanidins

The intake of food and nutrients was collected in the DHQ for each included participant. The DHQ is a 137-item self-administered food frequency questionnaire developed for evaluating the frequency and serving size of food consumed over the past year. The DHQ nutrient variables are calculated from the questionnaire responses by the DietCalc software, which takes into account food frequency, serving size, and other responses and uses these in conjunction with a nutrient database based on national dietary data (USDA 1994–1996 Continuing Survey of Food Intakes by Individuals (CSFII), available from the USDA Food Surveys Research Group, or the Nutrition Data Systems for Research (NDS-R) from the University of Minnesota, USA, which has nutrient values not available from the USDA Survey Nutrient Database) to calculate the daily intake of all nutrients in the database.

Anthocyanidins are one of the important subfamilies of flavonoids. Cyanidin, delphinidin, malvidin, peonidin, petunidin, and pelargonidin are the six common anthocyanidins [7,23]. In this study, the daily intake of cyanidin, delphinidin, malvidin, peonidin, petunidin, and pelargonidin was extracted from DHQ. The subclasses of anthocyanidins are “50%” variables. The amounts for processed foods were assumed to be 50% of the raw food for deriving the nutrient database values to account for losses due to processing. The total daily intake of anthocyanidins was the total sum of cyanidin, delphinidin, malvidin, pelargonidin, peonidin, and petunidin [7,8].

### 2.4. Lung Cancer Ascertainment

In this study, the outcome was the incidence of lung cancer. In the PLCO trial, the confirmation of the diagnosis of lung cancer was based on reports abstracted from the annual study update forms, and then ICD-O codes were used for extracting relevant medical records using standardized forms. Of note, carcinoid lung cancer was not considered as a target of lung cancer screening in the PLCO trial; thus, it was not confirmed as lung cancer in this study.

### 2.5. Statistical Analysis

Baseline characteristics of participants are presented as the quartile of total anthocyanidins (quartile 1 to quartile 4). Continuous variables are presented as mean (standard deviation), and categorical variables are presented as numbers (percentage). The Kruskal–Wallis test and chi-square test were used to compare continuous and categorical variables across the groups of participants, respectively. To test whether a trend across quartiles of anthocyanidins existed for the outcome, tests for trends across anthocyanidin quartiles were conducted by assigning each quartile the median value and treating the variable as a linear term in the regression models. Hazard ratios (HRs) and 95% confidence intervals (CIs) were calculated with Cox proportional hazards regression for the association of total anthocyanidins, cyanidin, delphinidin, malvidin, pelargonidin, peonidin, and petunidin and lung cancer incidence in all included participants and sex-specific groups, respectively. Sub-analyses were further performed to evaluate associations with different histological types, including adenocarcinoma, squamous cell carcinoma, large cell carcinoma, and small cell carcinoma. Covariates included in the multivariate regression models were based on the literature review and clinical judgement. In detail, age, gender, BMI, total energy intake, family history of lung cancer, marital status, race/ethnicity, cigarette pack-years, and alcohol intake were adjusted as covariates. The dose-response relationship was explored between total anthocyanidins and the incidence of lung cancer. We excluded the highest 5% of the value of total anthocyanidins in order to avoid the impact of outliers on the trend in the dose-response analysis. A restricted cubic spline model with three knots at the 10th, 50th, and 90th percentiles was employed [24]. We chose the median value of total anthocyanidins as the reference level [24]. Prespecified subgroup analyses were performed to evaluate whether the observed association of intake of total anthocyanidins with lung cancer incidence was modified by age (>65 vs. ≤65 years old), BMI (>25 vs. ≤25 kg/m^2^), family history of lung cancer (yes vs. no or possible), race/ethnicity (white vs. non-white) or smoking status (never vs. 0–20 pack-years vs. >20 pack-years). Effect modification by variables was examined by adding the cross-product of each effect modifier with total anthocyanidin quartiles in the multivariable-adjusted model. In addition, we conducted the sensitivity analysis to test the robustness of the results by excluding participants (1) with extreme energy intake (<800/>4000 kcal/day for males and <500/>3500 kcal/day for females) [25], (2) with the highest 1% intake of anthocyanidins (including total anthocyanidins, cyanidin, delphinidin, malvidin, peonidin, petunidin, and pelargonidin), or (3) with a follow-up less than 2 years. A two-tailed *p* value less than 0.05 was considered significant. The statistical analyses were conducted using STATA 15.1, SPSS 25.0, and R 3.6.1 software.

## 3. Results

### 3.1. Baseline Characteristics

**Figure 1:**
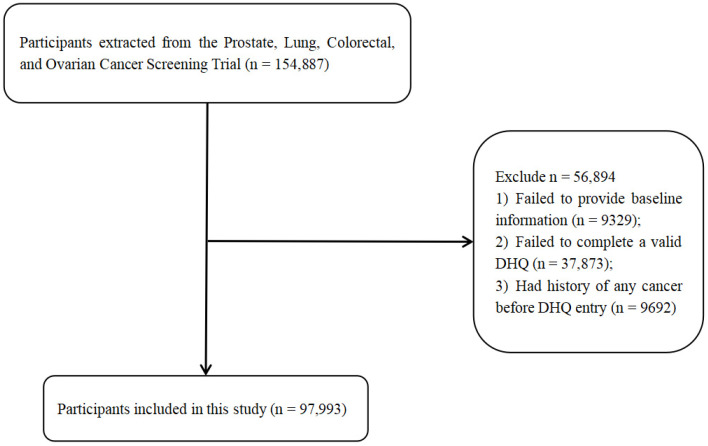
Flow diagram of the selected individuals.

Data of a total of 97,993 participants were extracted after excluding the participants according to exclusion criteria. The detailed flow chart is presented in Figure 1. We divided participants into quartiles of total anthocyanidins intake (24,533 in Q1; 24,467 in Q2; 24,516 in Q3; 24,477 in Q4). A total of 1631 lung cancer cases were obtained. There were 50,218 (51.25%) participants enrolled in the intervention group and 47,775 (48.75%) participants recruited to the control group. Significant differences were obtained in total energy intake, age, gender, BMI, race/ethnicity, marital status, cigarette pack-years, alcohol intake, family history of lung cancer, and family history of any cancer (all *p* < 0.05). In the highest quartile group of total anthocyanidins, participants had higher daily energy, were older, had lower cigarette pack-years, and had higher rates of clear family history of lung cancer. More detailed information is shown in Table 1.

### 3.2. Association between Anthocyanidins and Lung Cancer Risk

In the unadjusted analyses, the intakes of total anthocyanidins, cyanidin, delphinidin, malvidin, pelargonidin, peonidin, and petunidin were statistically significantly associated with at least a 40% reduction in the risk of lung cancer for comparison of the highest vs. lowest quartiles [(HR_Q4vsQ1_ for total anthocyanidins: 0.50; 95% CI: 0.44,0.58; *p* for trend < 0.001); (HR_Q4vsQ1_ for cyanidin: 0.55; 95% CI: 0.48,0.63; *p* for trend < 0.001); (HR_Q4vsQ1_ for delphinidin: 0.60; 95% CI: 0.53,0.69; *p* for trend <0.001); (HR_Q4vsQ1_ for malvidin: 0.54; 95% CI: 0.47,0.62; *p* for trend < 0.001); (HR_Q4vsQ1_ for pelargonidin: 0.52; 95% CI: 0.46,0.60; *p* for trend < 0.001); (HR_Q4vsQ1_ for peonidin: 0.53; 95% CI: 0.47,0.61; *p* for trend < 0.001); (HR_Q4vsQ1_ for petunidin: 0.60; 95% CI: 0.52,0.69; *p* for trend <0.001)]. In the multivariate adjusted regression model, the calculated adjusted HRs showed a trend that a higher quartile of total anthocyanidins, cyanidin, delphinidin, malvidin, pelargonidin, peonidin, and petunidin indicated lower risk of lung cancer [(HR_Q4vsQ1_ for total anthocyanidins: 0.63; 95% CI: 0.55,0.73; *p* for trend < 0.001); (HR_Q4vsQ1_ for cyanidin: 0.73; 95% CI: 0.63,0.84; *p* for trend < 0.001); (HR_Q4vsQ1_ for delphinidin: 0.70; 95% CI: 0.60,0.80; *p* for trend < 0.001); (HR_Q4vsQ1_ for malvidin: 0.65; 95% CI: 0.56,0.75; *p* for trend < 0.001); (HR_Q4vsQ1_ for pelargonidin: 0.75; 95% CI: 0.65,0.87; *p* for trend = 0.001); (HR_Q4vsQ1_ for peonidin: 0.63; 95% CI: 0.55,0.73; *p* for trend < 0.001); (HR_Q4vsQ1_ for petunidin: 0.70; 95% CI: 0.60,0.81; *p* for trend = 0.001)]. Overall, differences in the results by sex were not statistically significant for total anthocyanidins and the subclasses (all *p*-interaction due to sex > 0.05). However, there was a tendentious suggestion that the results differed between females and males for intakes of delphinidin, pelargonidin, and petunidin for comparison across quartiles. In men, higher intake of delphinidin (*p* for trend <0.001), pelargonidin (*p* for trend = 0.003), and petunidin (*p* for trend <0.001) was associated with a reduced risk of lung cancer, and a significant difference was not found in women (*p* for trend > 0.05) (Table 2).

We also examined the associations by lung cancer histologic type. In the unadjusted regression model, a significant inverse association between delphinidin and adenocarcinoma was not obtained (*p* for trend = 0.054). A significant inverse association between cyanidin and large cell carcinoma was also not obtained (*p* for trend = 0.072). However, other significant inverse associations between higher intakes of anthocyanidins and the risk of lung cancer cell types were observed. In the multivariate analyses, each of the associations was greatly attenuated when adjusted for potential covariates, for comparison of the highest vs. lowest quartiles. An inverse association between the intake of cyanidin, malvidin, and peonidin and the risk of adenocarcinoma was found, but not for total or other subclasses of anthocyanidins. An inverse association between the intake of total and subclasses of anthocyanidins and the risk of squamous cell carcinoma was observed. For large cell carcinoma, only the intake of malvidin was found to be associated with reduced cancer incidence. For small cell carcinoma, an inverse association was not found between the intake of delphinidin and the risk of small cell carcinoma. However, an association was found between total anthocyanidins and other subclasses of anthocyanidins and the risk of small cell carcinoma. Although a significant trend was not observed, the highest quartile of total anthocyanidins and pelargonidin was significantly associated with reduced risk of adenocarcinoma compared with the lowest quartile. The highest quartile of delphinidin was significantly associated with reduced risk of large cell carcinoma and small cell carcinoma compared with the lowest quartile. The highest quartile of peonidin was significantly associated with reduced risk of large cell carcinoma compared with the lowest quartile. Detailed information is shown in Table 3.

### 3.3. Additional Analyses

**Figure 2:**
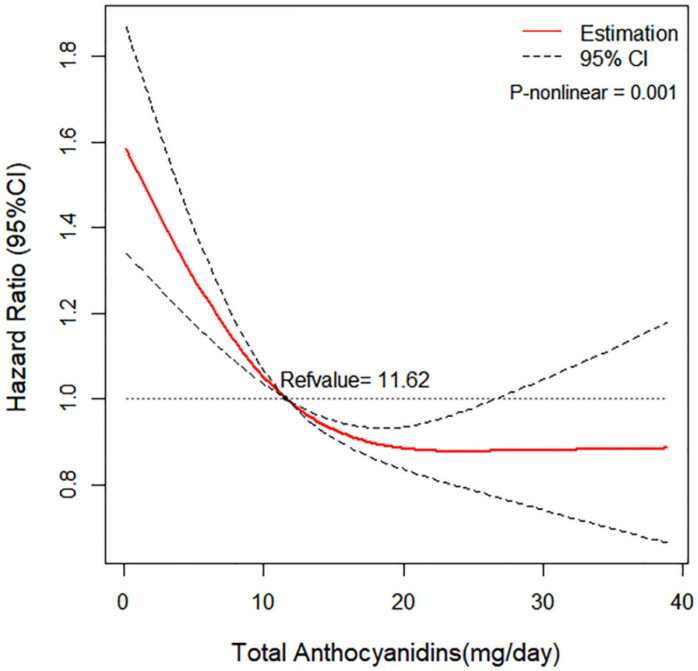
Dose-response relationship between anthocyanidins and risk of lung cancer adjusted for age, gender, BMI, total energy intake, family history of lung cancer, marital status, race/ethnicity, cigarette pack-years, and alcohol intake (*p* for non-linear = 0.001).

In order to explore the trend that the probability of lung cancer changed with the intake of total anthocyanidins, we conducted the dose-response analysis. A non-linear association between the intake of total anthocyanidins (reference value = 11.62 as median value) and lung cancer risk was found in the restricted cubic spline model (*p* for non-linear = 0.001) (Figure 2). The stratified analyses showed that the association between total anthocyanidins and lung cancer risk could be modified by smoking status (*p*-interaction = 0.004). The highest quartile of total anthocyanidins intake was associated with an elevated risk of lung cancer compared with the lowest quartile in non-smokers (HR_Q4vsQ1_: 2.18; 95% CI: 1.25,3.78) but associated with a reduced risk of lung cancer in former/current smokers with >20 cigarette pack-years (HR_Q4vsQ1_: 0.55; 95% CI: 0.47,0.65). The association between total anthocyanidins and risk of lung cancer differed in two age groups (*p*-interaction = 0.035). However, the association between total anthocyanidins intake and lung cancer risk was not found to be modified by BMI (>25 vs. ≤25 kg/m2), family history of lung cancer (yes vs. no or possible), or race/ethnicity (white vs. non-white) (Table 4). In sensitivity analyses, the HRs of total anthocyanidins did not change significantly by excluding participants with extreme energy intake, with the highest 1% intake of total anthocyanidins, or with a follow-up less than 2 years, indicating the robustness of the association between total and subclasses of anthocyanidins intake and the incidence of lung cancer (Table 5).

## 4. Discussion

This prospective large-scale cohort study suggests a reverse association between anthocyanidins and lung cancer risk in the American population. Dietary intake of total anthocyanidins and subclasses including cyanidin, malvidin, pelargonidin, peonidin, and petunidin are suggested to be related to a reduced risk of lung cancer after adjusting for potential confounding factors.

Differences in the results by sex were not statistically significant for anthocyanidins intake, though the tendentious association between certain subclasses of anthocyanidins and the risk of lung cancer was not significantly observed in the female group. In the analyses for different histologic types of lung cancer, we observed that the inverse association between total anthocyanidins and the risk of adenocarcinoma, squamous cell carcinoma, and small cell carcinoma was still significant in the highest quartile compared with the lowest quartile of total anthocyanidins. However, the association was not observed in large cell carcinoma. Whether the phenomenon is attributed to certain mechanism or the small number of cases needs further investigation. The dose-response analysis showed a non-linear relationship between total anthocyanidins and the risk of lung cancer. The lung cancer risk changed with the increased intake of total anthocyanidins in a non-linear manner. Increasing the intake of anthocyanidins may lead to a waning increase in the preventive effects of anthocyanidins on lung cancer. The waning increase in the preventive effects of anthocyanidins on lung cancer with the increasing intake of anthocyanidins indicates that the efficient intake of anthocyanidins against lung cancer may be no more than 20 mg/day according to the dose-response analysis. The stratified analyses suggest that the inverse association between total anthocyanidins and the risk of lung cancer could be modified by age and smoking status. In participants under 65 years old, the inverse association between total anthocyanidins and lung cancer risk was more clearly observed, compared with individuals older than 65 years. Moreover, high intake of total anthocyanidins may decrease the risk of lung cancer in heavy smokers of more than 20 cigarette pack-years. Previous evidence has shown that smokers have a high inflammatory response in the body, which is an important risk factor for lung cancer [26,27]. The evidence that anthocyanidins have anti-inflammatory effects [28] may explain why heavy smokers have opposite responses to anthocyanidins than non-smokers. Surprisingly, high intake of total anthocyanidins may increase the risk of lung cancer in non-smokers, whether anthocyanidins only have protective effect on lung cancer in people with high levels of inflammation merits further investigation. Sensitivity analyses indicated the robustness of the association between total anthocyanidins intake and the incidence of lung cancer.

Overall, this study shows differences from the previous two human studies mentioned above [18,19]. We notice that the daily intake of anthocyanidins in one previous study [18] was too small to compare with this study. And the other study did not describe the intake of anthocyanidins of participants in detail [19]. The concentration of anthocyanidins declines sharply in human blood through digestion. The attenuation of the concentration may induce inconspicuous anticancer effects to exert chemopreventive effects inhibiting the growth of malignant cells, inducing apoptosis and regulating carcinogenic signal transduction in human body [29]. Moreover, the biological activities of anthocyanidins were often influenced by intestinal absorption and mediated by microbial catabolites through habitual dietary intake, which decreases the effect of anthocyanidins in the human body [13]. Thus, low dietary intake of anthocyanidins may be not effectively biologically active in the human body [13]. Despite much lower intake of anthocyanidins than in this study, Cutler et al. suggested that there was a trend that a higher level of dietary anthocyanidins was associated with reduced risk of lung cancer in female ever-smokers (HR: 0.83; 95% CI: 0.67,1.01) compared with a lower intake of anthocyanidins [18]. It is also worth noting that the number of lung cancer cases in the previous two studies was relatively small and resulted in wider confidence intervals.

In summary, this study showed strengths and offers value for lung cancer prevention. This is the first large-scale prospective study exploring the association between the intake of anthocyanidins including total and subclasses and lung cancer risk in both females and males in the American population. We also evaluated the different associations for different genders and for different histologic types of lung cancer. Related potential confounding factors were adjusted to obtain more accurate results. Subgroup analyses evaluated factors that could modify the results, and consequently, we found that age and smoking status could significantly modify the relationship. In addition, the sensitivity analyses ascertained the robustness of the outcomes. Furthermore, the dose-response analysis showed the real relationship between the intake of total anthocyanidins and lung cancer risk in a visual way.

Flaws also exist in this study. Firstly, as dietary habits may change during the long-term follow-up, using only baseline diet to evaluate the dietary intake generally yielded weaker associations with the incidence of disease than using the cumulative dietary intake [30]. Secondly, confidence limits existed in the individual’s self-analysis regarding diet patterns, although the questionnaires were validated. Thirdly, the potential co-linearity between anthocyanidins and main food such as vitamins and fiber that may be responsible for the associations observed should be considered in future investigation.

## 5. Conclusions

In this study, we observed a protective association between dietary anthocyanidins and the risk of lung cancer in the American population. The lung cancer risk changed with the increase in intake of total anthocyanidins in a non-linear manner. Increasing intake of anthocyanidins (less than 20 mg/day) may lead to the waning increase in the preventive effects of anthocyanidins on lung cancer. Further studies should be conducted to confirm the association.

## Figures and Tables

**Table 1:** Baseline characteristics of 97,993 participants in the Prostate, Lung, Colorectal, and Ovarian (PLCO) Cancer Screening Trial, 1993–2009.

	Overall	Quartile 1	Quartile 2	Quartile 3	Quartile 4	*p*
**Number of participants**	97,993	24,533	24,467	24,516	24,477	-
**Number of cases**	1631	593	379	353	306	-
**Follow-up, years**	8.83 (1.96)	8.68 (2.03)	8.84 (1.97)	8.89 (1.92)	8.91 (1.90)	-
**Person-years**	865,382.70	213,064.20	216,317.80	217,948.80	218,051.90	-
**Total energy intake, kcal/day**	1738.34 (734.92)	1519.21 (712.76)	1652.74 (686.51)	1777.57 (698.68)	2004.25 (751.94)	<0.001
**Total anthocyanidins, mg/d**	15.86 (14.01)	4.34 (1.63)	9.48 (1.50)	15.74 (2.28)	33.91 (16.61)	<0.001
**Cyanidin, mg/d**	3.58 (3.88)	1.10 (0.68)	2.13 (1.16)	3.43 (1.85)	7.66 (5.49)	<0.001
**Delphinidin, mg/d**	4.50 (3.68)	1.32 (0.99)	3.44 (1.93)	5.09 (2.53)	8.14 (4.27)	<0.001
**Malvidin, mg/d**	4.04 (5.93)	0.86 (0.59)	1.82 (1.23)	3.62 (2.43)	9.85 (9.16)	<0.001
**Pelargonidin, mg/d**	2.56 (3.86)	0.77 (0.64)	1.49 (1.26)	2.53 (2.25)	5.45 (6.30)	<0.001
**Peonidin, mg/d**	0.50 (0.65)	0.11 (0.07)	0.23 (0.13)	0.44 (0.26)	1.20 (0.95)	<0.001
**Petunidin, mg/d**	0.69 (0.84)	0.19 (0.11)	0.35 (0.19)	0.63 (0.34)	1.61 (1.20)	<0.001
**Trial arm**						0.379
Intervention	50,218 (51.25%)	12,485 (50.89%)	12,492 (51.06%)	12,639 (51.55%)	12,602 (51.49%)	
Control	47,775 (48.75%)	12,048 (49.11%)	11,975 (48.94%)	11,877 (48.45%)	11,875 (48.51%)	
**Age, years**	65.50 (5.73)	64.90 (5.61)	65.58 (5.75)	65.76 (5.75)	65.76 (5.75)	<0.001
**Gender**						<0.001
Female	50,484 (51.52%)	10,559 (43.04%)	12,032 (49.18%)	13,160 (53.68%)	14,733 (60.19%)	
Male	47,509 (48.48%)	13,974 (56.96%)	12,435 (50.82%)	11,356 (46.32%)	9744 (39.81%)	
**Baseline body mass index, kg/m^2^**	27.22 (4.81)	27.59 (4.79)	27.43 (4.80)	27.06 (4.73)	26.81 (4.89)	<0.001
**Race/ethnicity**						<0.001
White	89,207 (91.03%)	21,904 (89.28%)	22,461 (91.80%)	22,624 (92.28%)	22,218 (90.77%)	
Non-white	8786 (8.97%)	2629 (10.72%)	2006 (8.20%)	1892 (7.72%)	2259 (9.23%)	
**Marital status**						<0.001
Married	76,847 (78.42%)	18,870 (76.92%)	19,395 (79.27%)	19,515 (79.60%)	19,067 (77.9%)	
Not married	21,146 (21.58%)	5663 (23.08%)	5072 (20.73%)	5001 (20.40%)	5410 (22.10%)	
**Cigarette pack-years**	17.85 (26.72)	24.20 (30.71)	17.58 (26.00)	15.31 (24.45)	14.31 (24.06)	<0.001
**Alcohol intake**						<0.001
Never	9910 (10.11%)	2414 (9.84%)	2587 (10.57%)	2440 (9.95%)	2469 (10.09%)	
Former	14,188 (14.48%)	4244 (17.30%)	3492 (14.27%)	3230 (13.18%)	3222 (13.16%)	
Current	71,151 (72.61%)	17,152 (69.91%)	17,684 (72.28%)	18,249 (74.44%)	18,066 (73.81%)	
Unknown	2744 (2.80%)	723 (2.95%)	704 (2.88%)	597 (2.44%)	720 (2.94%)	
**Family history of lung cancer**						<0.001
Yes	85,392 (87.14%)	21,148 (86.2%)	21,377 (87.37%)	21,418 (87.36%)	21,449 (87.63%)	
No	10,252 (10.46%)	2657 (10.83%)	2522 (10.31%)	2542 (10.37%)	2531 (10.34%)	
Possible	2349 (2.40%)	728 (2.97%)	568 (2.32%)	556 (2.27%)	497 (2.03%)	
**Family history of any cancer**						0.002
Yes	43,256 (44.14%)	11,031 (44.96%)	10,881 (44.47%)	10,679 (43.56%)	10,665 (43.57%)	
No	54,737 (55.86%)	13,502 (55.04%)	13,586 (55.53%)	13,837 (56.44%)	13,812 (56.43%)	

Note: Continuous variables were presented as mean (standard deviation), and categorical variables were presented as numbers (percentage).

**Table 2:** HRs (95% CIs) of lung cancer for quartiles of anthocyanidins.

	Quartile 1	Quartile 2	Quartile 3	Quartile 4	*p*-Trend	*p*-Interaction Due to Sex
**Total anthocyanidins**						
Unadjusted	1.00 (reference)	0.63 (0.55,0.71)	0.58 (0.51,0.66)	0.50 (0.44,0.58)	<0.001	
Multivariate *	1.00 (reference)	0.77 (0.67,0.88)	0.74 (0.65,0.85)	0.63 (0.55,0.73)	<0.001	0.691
Multivariate for female	1.00 (reference)	0.74 (0.60,0.93)	0.73 (0.59,0.91)	0.62 (0.50,0.77)	<0.001	
Multivariate for male	1.00 (reference)	0.79 (0.67,0.93)	0.75 (0.63,0.89)	0.63 (0.51,0.76)	<0.001	
**Cyanidin**						
Unadjusted	1.00 (reference)	0.67 (0.59,0.76)	0.47 (0.41,0.55)	0.55 (0.48,0.63)	<0.001	
Multivariate *	1.00 (reference)	0.81 (0.71,0.92)	0.62 (0.54,0.72)	0.73 (0.63,0.84)	<0.001	0.634
Multivariate for female	1.00 (reference)	0.76 (0.62,0.93)	0.61 (0.48,0.76)	0.70 (0.56,0.87)	<0.001	
Multivariate for male	1.00 (reference)	0.83 (0.70,0.97)	0.64 (0.53,0.77)	0.74 (0.62,0.90)	<0.001	
**Delphinidin**						
Unadjusted	1.00 (reference)	0.69 (0.61,0.79)	0.65 (0.57,0.75)	0.60 (0.53,0.69)	<0.001	
Multivariate *	1.00 (reference)	0.82 (0.72,0.94)	0.81 (0.70,0.92)	0.70 (0.60,0.80)	<0.001	0.214
Multivariate for female	1.00 (reference)	0.96 (0.78,1.19)	0.81 (0.65,1.01)	0.77 (0.61,0.96)	0.061	
Multivariate for male	1.00 (reference)	0.74 (0.62,0.88)	0.82 (0.69,0.97)	0.65 (0.54,0.78)	<0.001	
**Malvidin**						
Unadjusted	1.00 (reference)	0.66 (0.58,0.75)	0.56 (0.49,0.64)	0.54 (0.47,0.62)	<0.001	
Multivariate *	1.00 (reference)	0.78 (0.68,0.88)	0.70 (0.61,0.81)	0.65 (0.56,0.75)	<0.001	0.887
Multivariate for female	1.00 (reference)	0.81 (0.65,1.01)	0.79 (0.63,0.98)	0.71 (0.57,0.89)	0.02	
Multivariate for male	1.00 (reference)	0.76 (0.65,0.90)	0.66 (0.55,0.79)	0.61 (0.50,0.73)	<0.001	
**Pelargonidin**						
Unadjusted	1.00 (reference)	0.67 (0.59,0.77)	0.64 (0.56,0.72)	0.52 (0.46,0.60)	<0.001	
Multivariate *	1.00 (reference)	0.83 (0.73,0.95)	0.86 (0.75,0.99)	0.75 (0.65,0.87)	0.001	0.935
Multivariate for female	1.00 (reference)	0.91 (0.73,1.13)	0.92 (0.74,1.14)	0.80 (0.64,1.00)	0.256	
Multivariate for male	1.00 (reference)	0.79 (0.67,0.93)	0.83 (0.70,0.99)	0.73 (0.60,0.89)	0.003	
**Peonidin**						
Unadjusted	1.00 (reference)	0.65 (0.57,0.73)	0.57 (0.50,0.65)	0.53 (0.47,0.61)	<0.001	
Multivariate *	1.00 (reference)	0.76 (0.67,0.87)	0.70 (0.61,0.80)	0.63 (0.55,0.73)	<0.001	0.758
Multivariate for female	1.00 (reference)	0.76 (0.61,0.95)	0.77 (0.62,0.96)	0.66 (0.53,0.82)	0.002	
Multivariate for male	1.00 (reference)	0.77 (0.65,0.90)	0.66 (0.55,0.79)	0.61 (0.50,0.74)	<0.001	
**Petunidin**						
Unadjusted	1.00 (reference)	0.77 (0.68,0.88)	0.66 (0.58,0.76)	0.60 (0.52,0.69)	<0.001	
Multivariate *	1.00 (reference)	0.89 (0.78,1.01)	0.78 (0.68,0.89)	0.70 (0.60,0.81)	0.001	0.491
Multivariate for female	1.00 (reference)	0.89 (0.71,1.10)	0.73 (0.58,0.92)	0.73 (0.59,0.90)	0.124	
Multivariate for male	1.00 (reference)	0.89 (0.76,1.05)	0.81 (0.68,0.96)	0.66 (0.54,0.81)	0.001	

* adjusted for age (continuous), gender (male or female), BMI (continuous), total energy intake (continuous), family history of lung cancer (yes, no or possible), marital status (married or not married), race/ethnicity (white or non-white), cigarette pack-years (continuous), alcohol intake (never, former, current, or unknown).

**Table 3:** HRs of the association between anthocyanidins and incidence of lung cancer by histologic type.

	Adenocarcinoma				Squamous Cell Carcinoma				Large Cell Carcinoma				Small Cell Carcinoma			
	No. of Cases	Incidence Rate/10,000 Person-Years	Crude HR (95% CI)	Adjusted HR (95% CI) *	No. of Cases	Incidence Rate/10,000 Person-Years	Crude HR (95% CI)	Adjusted HR (95% CI) *	No. of Cases	Incidence Rate/10,000 Person-Years	Crude HR (95% CI)	Adjusted HR (95% CI) *	No. of Cases	Incidence Rate/10,000 Person-Years	Crude HR (95% CI)	Adjusted HR (95% CI) *
**Total anthocyanidins**																
	179	8.40	1.00 (reference)	1.00 (reference)	130	6.10	1.00 (reference)	1.00 (reference)	22	1.03	1.00 (reference)	1.00(reference)	85	3.99	1.00 (reference)	1.00 (reference)
	154	7.12	0.85 (0.68,1.05)	1.02 (0.82,1.26)	65	3.00	0.49 (0.37,0.66)	0.63 (0.47,0.85)	11	0.51	0.50 (0.24,1.02)	0.67 (0.32,1.39)	56	2.59	0.65 (0.46,0.91)	0.78 (0.55,1.10)
	148	6.79	0.81 (0.65,1.00)	1.01 (0.81,1.27)	66	3.03	0.50 (0.37,0.67)	0.69 (0.51,0.94)	9	0.41	0.40 (0.19,0.88)	0.58 (0.26,1.29)	47	2.16	0.54 (0.38,0.77)	0.67 (0.47,0.97)
	111	5.09	0.60 (0.48,0.77)	0.76 (0.59,0.97)	58	2.66	0.44 (0.32,0.59)	0.61 (0.44,0.84)	7	0.32	0.31 (0.13,0.74)	0.43 (0.18,1.05)	36	1.65	0.41 (0.28,0.61)	0.49 (0.32,0.73)
*p*-trend			0.001	0.072			<0.001	0.003			0.015	0.246			<0.001	0.005
**Cyanidin**																
	196	9.15	1.00 (reference)	1.00 (reference)	127	5.93	1.00 (reference)	1.00 (reference)	20	0.93	1.00 (reference)	1.00 (reference)	88	4.11	1.00 (reference)	1.00 (reference)
	151	6.96	0.76 (0.62,0.94)	0.90 (0.72,1.11)	69	3.18	0.54 (0.40,0.72)	0.68 (0.50,0.91)	12	0.55	0.60 (0.29,1.22)	0.76 (0.37,1.56)	57	2.63	0.64 (0.46,0.89)	0.76 (0.54,1.07)
	105	4.83	0.53 (0.42,0.67)	0.68 (0.53,0.87)	63	2.90	0.49 (0.36,0.66)	0.69 (0.51,0.94)	8	0.37	0.40 (0.18,0.90)	0.58 (0.25,1.35)	38	1.75	0.42 (0.29,0.62)	0.54 (0.36,0.80)
	140	6.46	0.71 (0.57,0.88)	0.93 (0.74,1.17)	60	2.77	0.47 (0.34,0.63)	0.67 (0.49,0.93)	9	0.42	0.45 (0.20,0.99)	0.61 (0.27,1.39)	41	1.89	0.46 (0.32,0.67)	0.57 (0.39,0.84)
*p*-trend			<0.001	0.016			<0.001	0.016			0.072	0.519			<0.001	0.004
**Delphinidin**																
	169	7.91	1.00 (reference)	1.00 (reference)	123	5.76	1.00 (reference)	1.00 (reference)	23	1.08	1.00 (reference)	1.00 (reference)	74	3.46	1.00 (reference)	1.00 (reference)
	149	6.90	0.87 (0.70,1.08)	1.02 (0.82,1.28)	66	3.06	0.53 (0.39,0.71)	0.65 (0.48,0.89)	10	0.46	0.43 (0.21,0.91)	0.56 (0.26,1.18)	48	2.22	0.64 (0.45,0.92)	0.74 (0.51,1.08)
	149	6.84	0.86 (0.69,1.07)	1.05 (0.84,1.32)	70	3.21	0.56 (0.42,0.75)	0.73 (0.54,0.98)	9	0.41	0.39 (0.18,0.84)	0.51 (0.23,1.13)	60	2.75	0.79 (0.56,1.11)	0.97 (0.68,1.38)
	125	5.74	0.72 (0.57,0.91)	0.85 (0.67,1.08)	60	2.75	0.48 (0.35,0.65)	0.58 (0.42,0.79)	7	0.32	0.30 (0.13,0.70)	0.34 (0.14,0.82)	42	1.93	0.55 (0.38,0.81)	0.63 (0.43,0.93)
*p*-trend			0.054	0.316			<0.001	0.003			0.007	0.066			0.01	0.061
**Malvidin**																
	191	8.81	1.00 (reference)	1.00 (reference)	122	5.63	1.00 (reference)	1.00 (reference)	25	1.15	1.00 (reference)	1.00 (reference)	84	3.88	1.00 (reference)	1.00 (reference)
	141	6.55	0.74 (0.60,0.92)	0.86 (0.69,1.08)	79	3.67	0.65 (0.49,0.86)	0.79 (0.60,1.06)	10	0.46	0.40 (0.19,0.84)	0.50 (0.24,1.05)	58	2.69	0.69 (0.50,0.97)	0.77 (0.55,1.08)
	145	6.71	0.76 (0.61,0.94)	0.92 (0.74,1.15)	54	2.50	0.44 (0.32,0.61)	0.59 (0.42,0.82)	10	0.46	0.40 (0.19,0.84)	0.54 (0.26,1.15)	42	1.94	0.50 (0.35,0.73)	0.59 (0.40,0.86)
	115	5.29	0.60 (0.48,0.76)	0.70 (0.55,0.90)	64	2.94	0.52 (0.39,0.71)	0.69 (0.51,0.95)	4	0.18	0.16 (0.06,0.46)	0.21 (0.07,0.62)	40	1.84	0.47 (0.33,0.69)	0.53 (0.36,0.79)
*p*-trend			<0.001	0.035			<0.001	0.01			0.001	0.019			<0.001	0.005
**Pelargonidin**																
	208	9.64	1.00 (reference)	1.00 (reference)	118	5.47	1.00 (reference)	1.00 (reference)	21	0.97	1.00 (reference)	1.00 (reference)	88	4.08	1.00 (reference)	1.00 (reference)
	140	6.49	0.67 (0.54,0.83)	0.81 (0.65,1.00)	62	2.87	0.52 (0.39,0.71)	0.69 (0.51,0.94)	11	0.51	0.53 (0.25,1.09)	0.75 (0.36,1.59)	47	2.18	0.53 (0.38,0.76)	0.64 (0.44,0.91)
	126	5.81	0.60 (0.48,0.75)	0.78 (0.62,0.98)	83	3.83	0.70 (0.53,0.93)	1.06 (0.79,1.42)	11	0.51	0.52 (0.25,1.08)	0.87 (0.41,1.87)	54	2.49	0.61 (0.44,0.86)	0.79 (0.56,1.12)
	118	5.43	0.56 (0.45,0.71)	0.77 (0.60,0.97)	56	2.58	0.47 (0.34,0.65)	0.78 (0.56,1.09)	6	0.28	0.29 (0.12,0.71)	0.54 (0.21,1.38)	35	1.61	0.40 (0.27,0.58)	0.52 (0.34,0.78)
*p*-trend			<0.001	0.064			<0.001	0.03			0.029	0.607			<0.001	0.006
**Peonidin**																
	203	9.12	1.00 (reference)	1.00 (reference)	126	5.66	1.00 (reference)	1.00 (reference)	23	1.03	1.00 (reference)	1.00 (reference)	88	3.95	1.00 (reference)	1.00 (reference)
	130	6.04	0.66 (0.53,0.83)	0.77 (0.62,0.96)	79	3.67	0.65 (0.49,0.86)	0.79 (0.59,1.05)	10	0.46	0.45 (0.22,0.95)	0.56 (0.26,1.18)	58	2.70	0.68 (0.49,0.95)	0.77 (0.55,1.07)
	142	6.75	0.74 (0.60,0.92)	0.89 (0.71,1.11)	50	2.38	0.42 (0.30,0.58)	0.54 (0.39,0.76)	10	0.48	0.46 (0.22,0.97)	0.63 (0.29,1.35)	41	1.95	0.49 (0.34,0.71)	0.55 (0.38,0.81)
	117	5.38	0.59 (0.47,0.74)	0.69 (0.54,0.87)	64	2.95	0.52 (0.38,0.70)	0.68 (0.50,0.93)	6	0.28	0.27 (0.11,0.66)	0.35 (0.14,0.87)	37	1.70	0.43 (0.29,0.63)	0.47 (0.32,0.71)
*p*-trend			<0.001	0.01			<0.001	0.002			0.01	0.109			<0.001	0.001
**Petunidin**																
	181	8.10	1.00 (reference)	1.00 (reference)	114	5.10	1.00 (reference)	1.00 (reference)	21	0.94	1.00 (reference)	1.00 (reference)	85	3.80	1.00 (reference)	1.00 (reference)
	128	6.11	0.75 (0.60,0.94)	0.86 (0.68,1.08)	89	4.25	0.83 (0.63,1.10)	0.97 (0.73,1.28)	12	0.57	0.61 (0.30,1.24)	0.74 (0.36,1.52)	57	2.72	0.71 (0.51,1.00)	0.77 (0.55,1.08)
	153	7.09	0.87 (0.70,1.08)	1.03 (0.83,1.29)	53	2.45	0.48 (0.35,0.67)	0.58 (0.41,0.81)	9	0.42	0.45 (0.20,0.97)	0.57 (0.25,1.26)	44	2.04	0.54 (0.37,0.77)	0.56 (0.38,0.81)
	130	6.00	0.74 (0.59,0.93)	0.87 (0.68,1.10)	63	2.91	0.57 (0.42,0.78)	0.71 (0.51,0.98)	7	0.32	0.35 (0.15,0.81)	0.42 (0.17,1.02)	38	1.76	0.46 (0.31,0.68)	0.48 (0.32,0.72)
*p*-trend			0.026	0.283			<0.001	0.004			0.046	0.225			<0.001	0.001

* adjusted for age (continuous), gender (male or female), BMI (continuous), total energy intake (continuous), family history of lung cancer (yes, no or possible), marital status (married or not married), race/ethnicity (white or non-white), cigarette pack-years (continuous), alcohol intake (never, former, current, or unknown).

**Table 4:** Subgroup analyses on the association of total anthocyanidins with the risk of lung cancer.

Subgroup	Cases	Pearson-Years	HR (95% CI) *	*p*-Interaction
			Quartile 4 vs. Quartile 1	
**Age, years**				0.035
>65	1033	411,157	0.72 (0.60,0.87)	
≤65	598	454,225.7	0.58 (0.46,0.74)	
**BMI, kg/m^2^**				0.895
>25	995	564,941.1	0.61 (0.50,0.74)	
≤25	636	300,441.6	0.66 (0.53,0.83)	
**Race**				0.287
White	1510	788,776.1	0.62 (0.53,0.72)	
Non-White	121	76,606.64	0.81 (0.48,1.37)	
**Family history of lung cancer**				0.223
Yes	1285	755,559.2	0.51 (0.35,0.73)	
No/Possible	346	109,823.5	0.66 (0.56,0.77)	
**Smoking status**				0.004
Never	138	424,755.3	2.18 (1.25,3.78)	
0–20 pack-years	129	167,552.4	0.97 (0.58,1.61)	
≥20 pack-years	1364	273,075.1	0.55 (0.47,0.65)	

* adjusted for age (continuous), gender (male or female), BMI (continuous), total energy intake (continuous), family history of lung cancer (yes, no or possible), marital status (married or not married), race/ethnicity (white or non-white), cigarette pack-years (continuous), alcohol intake (never, former, current, or unknown).

**Table 5:** Sensitivity analyses to assess the robustness of the association between anthocyanidins and lung cancer.

	Adjusted HR (95% CI) * (Q4 vs. Q1)						
	Total Anthocyanidins	Cyanidin	Delphinidin	Malvidin	Pelargonidin	Peonidin	Petunidin
Primary analysis	0.63 (0.55, 0.73)	0.73 (0.63, 0.84)	0.70 (0.60, 0.80)	0.65 (0.56, 0.75)	0.75 (0.65, 0.87)	0.63 (0.55, 0.73)	0.70 (0.60, 0.81)
Excluding participants with extreme energy intake	0.66 (0.57, 0.76)	0.75 (0.65, 0.87)	0.72 (0.62, 0.83)	0.67 (0.58, 0.77)	0.78 (0.67, 0.90)	0.66 (0.57, 0.76)	0.72 (0.62, 0.84)
Excluding participants with the highest 1% intake of anthocyanidins	0.64 (0.55, 0.74)	0.74 (0.64, 0.85)	0.71 (0.61, 0.81)	0.66 (0.57, 0.76)	0.76 (0.65, 0.88)	0.64 (0.56, 0.74)	0.71 (0.61, 0.82)
Excluding participants with a follow-up less than 2 years	0.59 (0.50, 0.69)	0.68 (0.58, 0.80)	0.67 (0.58, 0.79)	0.62 (0.53, 0.72)	0.72 (0.62, 0.85)	0.61 (0.52, 0.71)	0.66 (0.57, 0.78)

* adjusted for age (continuous), gender (male or female), BMI (continuous), total energy intake (continuous), family history of lung cancer (yes, no or possible), marital status (married or not married), race/ethnicity (white or non-white), cigarette pack-years (continuous), alcohol intake (never, former, current, or unknown).

## Data Availability

Data that support the findings of this study have been deposited in the PLCO trial (https://biometry.nci.nih.gov/cdas/plco/ accessed on 2 March 2022) upon reasonable request.

## References

[1] GBD 2019 Diseases and Injuries Collaborators (2020). Global burden of 369 diseases and injuries in 204 countries and territories, 1990–2019: A systematic analysis for the Global Burden of Disease Study 2019. Lancet.

[2] Sung H., Ferlay J., Siegel R.L., Laversanne M., Soerjomataram I., Jemal A., Bray F. (2021). Global Cancer Statistics 2020: GLOBOCAN Estimates of Incidence and Mortality Worldwide for 36 Cancers in 185 Countries. CA Cancer J. Clin..

[3] Wei X., Zhu C., Ji M., Fan J., Xie J., Huang Y., Jiang X., Xu J., Yin R., Du L. (2021). Diet and Risk of Incident Lung Cancer: A Large Prospective Cohort Study in UK Biobank. Am. J. Clin. Nutr..

[4] Park S.-Y., Boushey C., Shvetsov Y., Wirth M., Shivappa N., Hébert J., Haiman C., Wilkens L., Le Marchand L. (2021). Diet Quality and Risk of Lung Cancer in the Multiethnic Cohort Study. Nutrients.

[5] Myneni A.A., Giovino G.A., Millen A.E., LaMonte M.J., Wactawski-Wende J., Neuhouser M.L., Zhao J., Shikany J.M., Mu L. (2021). Indices of Diet Quality and Risk of Lung Cancer in the Women’s Health Initiative Observational Study. J. Nutr..

[6] Pieńkowska N., Bartosz G., Furdak P., Sadowska-Bartosz I. (2021). Delphinidin Increases the Sensitivity of Ovarian Cancer Cell Lines to 3-Bromopyruvate. Int. J. Mol. Sci..

[7] Khoo H.E., Azlan A., Tang S.T., Lim S.M. (2017). Anthocyanidins and anthocyanins: Colored pigments as food, pharmaceutical ingredients, and the potential health benefits. Food Nutr. Res..

[8] Harnly J.M., Doherty R.F., Beecher G.R., Holden J.M., Haytowitz D.B., Bhagwat A.S., Gebhardt S. (2006). Flavonoid content of U.S. fruits, vegetables, and nuts. J. Agric. Food Chem..

[9] Chen Z., Zhang R., Shi W., Li L., Liu H., Liu Z., Wu L. (2019). The Multifunctional Benefits of Naturally Occurring Delphinidin and Its Glycosides. J. Agric. Food Chem..

[10] Park M., Sharma A., Lee H.J. (2019). Anti-Adipogenic Effects of Delphinidin-3-O-β-Glucoside in 3T3-L1 Preadipocytes and Primary White Adipocytes. Molecules.

[11] Hair R., Sakaki J.R., Chun O.K. (2021). Anthocyanins, Microbiome and Health Benefits in Aging. Molecules.

[12] Sharma A., Choi H.K., Kim Y.K., Lee H.J. (2021). Delphinidin and Its Glycosides’ War on Cancer: Preclinical Perspectives. Int. J. Mol. Sci..

[13] Wang L.S., Stoner G.D. (2008). Anthocyanins and their role in cancer prevention. Cancer Lett..

[14] Bars-Cortina D., Sakhawat A., Piñol-Felis C., Motilva M.J. (2022). Chemopreventive effects of anthocyanins on colorectal and breast cancer: A review. Semin. Cancer Biol..

[15] Dharmawansa K.V.S., Hoskin D.W., Rupasinghe H.P.V. (2020). Chemopreventive Effect of Dietary Anthocyanins against Gastrointestinal Cancers: A Review of Recent Advances and Perspectives. Int. J. Mol. Sci..

[16] Ding M., Feng R., Wang S.Y., Bowman L., Lu Y., Qian Y., Castranova V., Jiang B.-H., Shi X. (2006). Cyanidin-3-glucoside, a natural product derived from blackberry, exhibits chemopreventive and chemotherapeutic activity. J. Biol. Chem..

[17] Chen P.N., Chu S.C., Chiou H.L., Chiang C.L., Yang S.F., Hsieh Y.S. (2005). Cyanidin 3-glucoside and peonidin 3-glucoside inhibit tumor cell growth and induce apoptosis in vitro and suppress tumor growth in vivo. Nutr. Cancer.

[18] Cutler G.J., Nettleton J.A., Ross J.A., Harnack L.J., Jacobs D.R., Scrafford C.G., Barraj L.M., Mink P.J., Robien K. (2008). Dietary flavonoid intake and risk of cancer in postmenopausal women: The Iowa Women’s Health Study. Int. J. Cancer.

[19] Mursu J., Nurmi T., Tuomainen T.P., Salonen J.T., Pukkala E., Voutilainen S. (2008). Intake of flavonoids and risk of cancer in Finnish men: The Kuopio Ischaemic Heart Disease Risk Factor Study. Int. J. Cancer.

[20] Prorok P.C., Andriole G.L., Bresalier R., Buys S.S., Chia D., Crawford E.D., Fogel R., Gelmann E.P., Gilbert F., Hasson M.A. (2000). Design of the Prostate, Lung, Colorectal and Ovarian (PLCO) Cancer Screening Trial. Control. Clin. Trials.

[21] Thompson F.E., Subar A.F., Brown C.C., Smith A.F., Sharbaugh C.O., Jobe J.B., Mittl B., Gibson J.T., Ziegler R.G. (2002). Cognitive research enhances accuracy of food frequency questionnaire reports: Results of an experimental validation study. J. Am. Diet. Assoc..

[22] Subar A.F., Thompson F.E., Kipnis V., Midthune D., Hurwitz P., McNutt S., McIntosh A., Rosenfeld S. (2001). Comparative validation of the Block, Willett, and National Cancer Institute food frequency questionnaires: The Eating at America’s Table Study. Am. J. Epidemiol..

[23] He J., Giusti M.M. (2010). Anthocyanins: Natural colorants with health-promoting properties. Annu. Rev. Food Sci. Technol..

[24] Desquilbet L., Mariotti F. (2010). Dose-response analyses using restricted cubic spline functions in public health research. Stat. Med..

[25] Willett W. (2012). Nutritional Epidemiology.

[26] Caliri A.W., Tommasi S., Besaratinia A. (2021). Relationships among smoking, oxidative stress, inflammation, macromolecular damage, and cancer. Mutat. Res. Rev. Mutat. Res..

[27] Shields P.G., Berman M., Brasky T.M., Freudenheim J.L., Mathe E., McElroy J.P., Song M.A., Wewers M.D. (2017). A Review of Pulmonary Toxicity of Electronic Cigarettes in the Context of Smoking: A Focus on Inflammation. Cancer Epidemiol. Biomark. Prev..

[28] Kwon J.Y., Lee K.W., Hur H.J., Lee H.J. (2007). Peonidin inhibits phorbol-ester-induced COX-2 expression and transformation in JB6 P+ cells by blocking phosphorylation of ERK-1 and -2. Ann. N. Y. Acad. Sci..

[29] Yang M., Koo S.I., Song W.O., Chun O.K. (2011). Food matrix affecting anthocyanin bioavailability: Review. Curr. Med. Chem..

[30] Hu F.B., Stampfer M.J., Rimm E., Ascherio A., Rosner B.A., Spiegelman D., Willett W.C. (1999). Dietary fat and coronary heart disease: A comparison of approaches for adjusting for total energy intake and modeling repeated dietary measurements. Am. J. Epidemiol..

